# Systemic inflammatory markers are associated with neoadjuvant chemotherapy selection but not survival in advanced serous epithelial ovarian cancer

**DOI:** 10.1007/s11845-026-04407-x

**Published:** 2026-04-20

**Authors:** Yakup Yalcin, Kemal Ozerkan

**Affiliations:** Department of Obstetrics and Gynecology, School of Medicine, Bursa Uludag University, Bursa, 16110 Turkey

**Keywords:** Serous epithelial ovarian cancer, Systemic inflammatory markers, Neutrophil-to-lymphocyte ratio, Platelet-to-lymphocyte ratio, Systemic immune-inflammation index, Survival

## Abstract

**Background:**

Systemic inflammation plays an important role in ovarian cancer progression and has been associated with adverse outcomes. Neutrophil-lymphocyte ratio (NLR), platelet-lymphocyte ratio (PLR), and systemic immune-inflammatory index (SII) are markers that assess systemic inflammation.

**Aim:**

To evaluate the association between preoperative systemic inflammatory markers and the selection of neoadjuvant chemotherapy (NACT), and to assess their prognostic impact on overall survival (OS) and progression-free survival (PFS) in patients with advanced-stage serous epithelial ovarian cancer.

**Methods:**

This retrospective cohort study included patients with FIGO stage III–IV high-grade serous epithelial ovarian cancer. Patients underwent either primary debulking surgery (PDS) followed by platinum-based chemotherapy or NACT followed by interval debulking surgery. Preoperative inflammatory markers (NLR, PLR, and SII) were calculated and dichotomized using median cut-off values (NLR ≥ 4.30, PLR ≥ 222, SII ≥ 1350).

**Results:**

A total of 239 patients were included; 108 (45.1%) underwent PDS and 131 (54.9%) received NACT. Patients selected for NACT had significantly higher preoperative NLR, PLR, and SII values. In multivariable analyses, high NLR (OR 3.31) and high SII (OR 1.84) were independently associated with increased likelihood of NACT selection, whereas PLR was not. In multivariable analysis, none of the elevated inflammatory markers remained independently associated with prognosis. NACT was associated with inferior OS and PFS after multivariable adjustment. Kaplan–Meier analyses showed that inflammatory markers stratified survival in the PDS group but not in the NACT group.

**Conclusion:**

Preoperative systemic inflammatory markers, particularly NLR and SII, are strongly associated with the selection of neoadjuvant chemotherapy in advanced-stage serous epithelial ovarian cancer but do not independently predict survival outcomes.

## Introduction

Serous epithelial ovarian cancer represents the most common and aggressive subtype of epithelial ovarian cancer, accounting for approximately 70–80% of cases and the majority of ovarian cancer–related deaths [1, 2]. High-grade serous ovarian carcinoma (HGSOC) is typically diagnosed at an advanced stage (FIGO stage III–IV) due to its insidious onset and lack of specific early symptoms, resulting in poor long-term survival outcomes [3].

In advanced serous ovarian cancer, the standard therapeutic approach consists of primary debulking surgery (PDS) aiming at complete cytoreduction with no macroscopic residual disease, followed by platinum-based chemotherapy. The extent of residual disease after surgery is widely recognized as the most powerful prognostic factor influencing survival [4–6]. However, optimal cytoreduction cannot be achieved in all patients because of extensive peritoneal disease, high tumor burden, poor performance status, or significant comorbidities. In such cases, neoadjuvant chemotherapy (NACT) followed by interval debulking surgery (IDS) is considered an alternative treatment strategy [7, 8]. Although randomized phase III trials have demonstrated non-inferiority of NACT followed by IDS compared with PDS in terms of overall survival, real-world data suggest that patients selected for NACT generally represent a higher-risk population with more aggressive disease biology and worse outcomes [9–11]. Therefore, accurate identification of patients who are more likely to benefit from upfront surgery versus NACT remains a critical clinical challenge in serous epithelial ovarian cancer.

Systemic inflammation has emerged as a key component of cancer progression, tumor–host interaction, and response to therapy [12, 13]. In serous ovarian cancer, inflammatory and immune responses have been shown to correlate with tumor burden, metastatic potential, and treatment resistance. Easily accessible blood-based inflammatory markers derived from routine complete blood counts—such as the neutrophil-to-lymphocyte ratio (NLR), platelet-to-lymphocyte ratio (PLR), and systemic immune-inflammation index (SII)—have been widely investigated as prognostic biomarkers in epithelial ovarian cancer [14–18]. Elevated levels of these markers have consistently been associated with aggressive tumor behavior and poor overall survival (OS) and progression-free survival (PFS) in multiple retrospective studies and meta-analyses [19–21]. Nevertheless, most available evidence focuses on the prognostic value of inflammatory markers, while their potential role in guiding treatment selection—particularly the decision between PDS and NACT—has not been adequately explored. Moreover, it remains unclear whether these markers independently influence survival outcomes or primarily reflect disease burden and patient characteristics that drive treatment allocation.

The aim of the present study was to evaluate the association between preoperative systemic inflammatory markers (NLR, PLR, and SII) and the selection of neoadjuvant chemotherapy in patients with advanced-stage serous epithelial ovarian cancer. In addition, we investigated the impact of these markers on overall and progression-free survival, with stratified analyses according to treatment strategy, in order to clarify whether inflammatory markers serve as independent prognostic factors or rather as biomarkers reflecting treatment selection.

## Methods

### Study design and patient population

This retrospective cohort study included patients with advanced-stage serous epithelial ovarian cancer treated at a tertiary gynecologic oncology center between January 2010 and August 2025. Eligible patients were diagnosed with FIGO stage III or IV disease and received either PDS followed by platinum-based chemotherapy or NACT followed by IDS. Only patients with histologically confirmed high-grade serous epithelial ovarian carcinoma were included. Patients with non-serous histology, suboptimal surgery (residual tumor greater than 1 cm), Eastern Cooperative Oncology Group (ECOG) levels 3 and 4, borderline tumors, synchronous malignancies, active infections, autoimmune or inflammatory diseases, hematologic disorders, or incomplete clinical or laboratory data were excluded.

The study was conducted in accordance with the Declaration of Helsinki and approved by the local institutional ethics committee (Protocol No: 2025/854/16 − 5). Due to the retrospective design, informed consent was waived.

## Data collection

Clinical, pathological, and laboratory data were retrieved from electronic medical records. Collected variables included age at diagnosis, body mass index (BMI), ECOG performance status, FIGO stage, preoperative serum CA-125 levels, treatment strategy (PDS vs. NACT), residual disease status after surgery, and survival outcomes. Residual disease was categorized as no visible residual disease or residual disease *≤* 1 cm, in accordance with institutional practice.

## Laboratory measurements and inflammatory markers

Preoperative laboratory values were obtained from peripheral blood samples collected within 7 days before initiation of treatment (PDS or first cycle of NACT). Absolute neutrophil, lymphocyte, monocyte, and platelet counts were recorded.

Inflammatory markers were calculated as follows:

- Neutrophil-to-lymphocyte ratio (NLR): neutrophil count / lymphocyte count.
- Platelet-to-lymphocyte ratio (PLR): platelet count / lymphocyte count.
- Monocyte-to-lymphocyte ratio (MLR): monocyte count / lymphocyte count.
- Systemic immune-inflammation index (SII): platelet count × neutrophil count / lymphocyte count.

For regression analyses evaluating treatment selection, inflammatory markers were dichotomized using median cut-off values: NLR ≥ 4.30, PLR ≥ 222, and SII ≥ 1350. Due to collinearity among inflammatory markers, separate multivariable models were constructed for each marker.

## Outcomes

The primary outcome was the association between preoperative inflammatory markers and selection of neoadjuvant chemotherapy. Secondary outcomes included overall survival (OS) and progression-free survival (PFS). OS was defined as the time from diagnosis to death from any cause or last follow-up. PFS was defined as the time from diagnosis to disease progression or recurrence.

### Statistical analysis

All statistical analyses were performed using IBM SPSS Statistics for Windows, version 26.0 (IBM Corp., Armonk, NY, USA). Continuous variables were expressed as medians with ranges and compared using the Mann–Whitney U test. Categorical variables were presented as frequencies and percentages and compared using the χ² test or Fisher’s exact test, as appropriate.

To identify factors associated with the selection of NACT, multivariable logistic regression analyses were performed. Odds ratios (ORs) with 95% confidence intervals (CIs) were reported. Each inflammatory marker (NLR, PLR, and SII) was evaluated in a separate model adjusted for age, ECOG performance status, FIGO stage, and CA-125 level. This approach was adopted due to collinearity among inflammatory markers, as demonstrated by correlation analysis and variance inflation factor (VIF) assessment.

Survival outcomes, including OS and PFS, were analyzed using the Kaplan–Meier method and compared with the log-rank test. Cox proportional hazards regression models were used for univariable and multivariable survival analyses. Variables with *p* < 0.10 in univariable analyses, along with clinically relevant covariates, were included in multivariable models. Inflammatory markers were entered into separate Cox models to account for collinearity. Residual disease was not included in the primary multivariable survival models, as it represents a post-surgical variable closely linked to treatment strategy and may lie on the causal pathway between treatment and survival outcomes. Inclusion of this variable could lead to overadjustment and obscure the independent effects of pre-treatment factors, particularly systemic inflammatory markers.

All statistical tests were two-sided, and a p value < 0.05 was considered statistically significant.

## Results

### Demographic details

Baseline demographic, clinical, and laboratory characteristics of the study population are presented in Table 1. A total of 239 patients with advanced-stage serous epithelial ovarian cancer were included, of whom 108 (45.1%) underwent PDS and 131 (54.9%) received NACT. Patients in the NACT group were significantly older than those in the PDS group (median age 62 vs. 57 years, *p* = 0.046). Body mass index did not differ significantly between groups (*p* = 0.103). ECOG performance status distribution was comparable between PDS and NACT patients (*p* = 0.201), as was FIGO stage distribution, with stage IV disease observed in 8.3% of PDS patients and 11.5% of NACT patients (*p* = 0.561).

**Table 1:** Comparison of clinical and laboratory characteristics between PDS and NACT groups

Variables	Total *N*: 239	PDS *N*: 108 (45.1%)	NACT *N*: 131 (54.9%)	*p* value
Median age in y (range)	60 (34–86)	57 (34–86)	62 (35–83)	**0.046**
BMI (range)	27 (18–44.3)	26 (18.4–44)	28 (18–44.3)	0.103
ECOG, *n* (%) 0 1 2	157 (65.7%) 69 (28.9%) 13 (5.4%)	74 (68.5%) 26 (24.1%) 8 (7.4%)	83 (63.4%) 43 (32.8%) 5 (3.8%)	0.201
FIGO Stage, *n* (%) III IV	215 (90.0%) 24 (10.0%)	99 (91.7%) 9 (8.3%)	116 (88.5%) 15 (11.5%)	0.561
Median neutrophil count (x10^3^, range)	6.30 (1.90–19.60)	5.60 (1.90–14.00)	8.60 (1.90–19.60)	**< 0.001**
Median lymphocyte count (x10^3^, range)	1.50 (0.30–4.10)	1.60 (0.70–4.10)	1.50 (0.30–3.50)	0.090
Median monocyte count (x10^3^, range)	0.50 (0.10–2.10)	0.60 (0.10–1.20)	0.50 (0.10–2.10)	0.501
Median platelet count (x10^3^, range)	343 (79–751)	331 (97–684)	365 (79–751)	0.250
Median NLR (range)	4.30 (0.90–26.43)	3.43 (1.10–20.00)	5.26 (0.90–26.43)	**< 0.001**
Median PLR (range)	222 (49.3–997.1)	206.3 (74.2–734)	236 (49.3–997.1)	**0.032**
Median MLR (range)	0.33 (0.05–3.17)	0.31 (0.05–1.57)	0.36 (0.06–3.17)	0.175
Median SII (range)	1350 (158–18447)	1128 (230–5609)	1997 (158–18447)	**0.002**
Median CA125 (range)	427 (3–10000)	432 (7–10000)	410 (3–10000)	0.661
Median Albumin (range)	38 (11–52)	36.5 (11–49)	38 (21–52)	0.279
Median CRP (range)	2 (0.3–86)	2 (0.3–46)	2 (0.4–86)	0.223
Residual disease, *n* (%) Non-visible *≤*1 cm	170 (71.1%) 69 (28.9%)	79 (73.1%) 29 (26.9%)	91 (69.5%) 40 (30.5%)	0.630

Abbreviations**: ***PDS *primary debulking surgery, *NACT *neoadjuvant chemotherapy, *BMI *body mass index, *ECOG *Eastern Cooperative Oncology Group, *FIGO *International Federation of Gynecology and Obstetrics, *NLR *neutrophil-to-lymphocyte ratio, *PLR *platelet-to-lymphocyte ratio, *MLR *monocyte-to-lymphocyte ratio, *SII *systemic immune-inflammation index, *CA 125 *cancer antigen 125, *CRP *C-reactive protein

### Inflammatory and laboratory parameters

As shown in Table 1, preoperative inflammatory parameters differed significantly according to treatment strategy. Median neutrophil count was higher in the NACT group compared with the PDS group (8.60 vs. 5.60 × 10³/µL, *p* < 0.001), whereas lymphocyte (*p* = 0.090), monocyte (*p* = 0.501), and platelet counts (*p* = 0.250) did not differ significantly. Composite inflammatory indices were consistently elevated in patients selected for NACT. Median NLR was significantly higher in the NACT group compared with the PDS group (5.26 vs. 3.43, *p* < 0.001). Similarly, median PLR was higher in NACT patients (236 vs. 206.3, *p* = 0.032), and median SII was markedly increased in the NACT group (1997 vs. 1128, *p* = 0.002). Serum CA-125 (*p* = 0.661), albumin (*p* = 0.279), and C-reactive protein (*p* = 0.223) levels were comparable between treatment groups.

### Factors associated with neoadjuvant chemotherapy selection

**Fig. 1:**
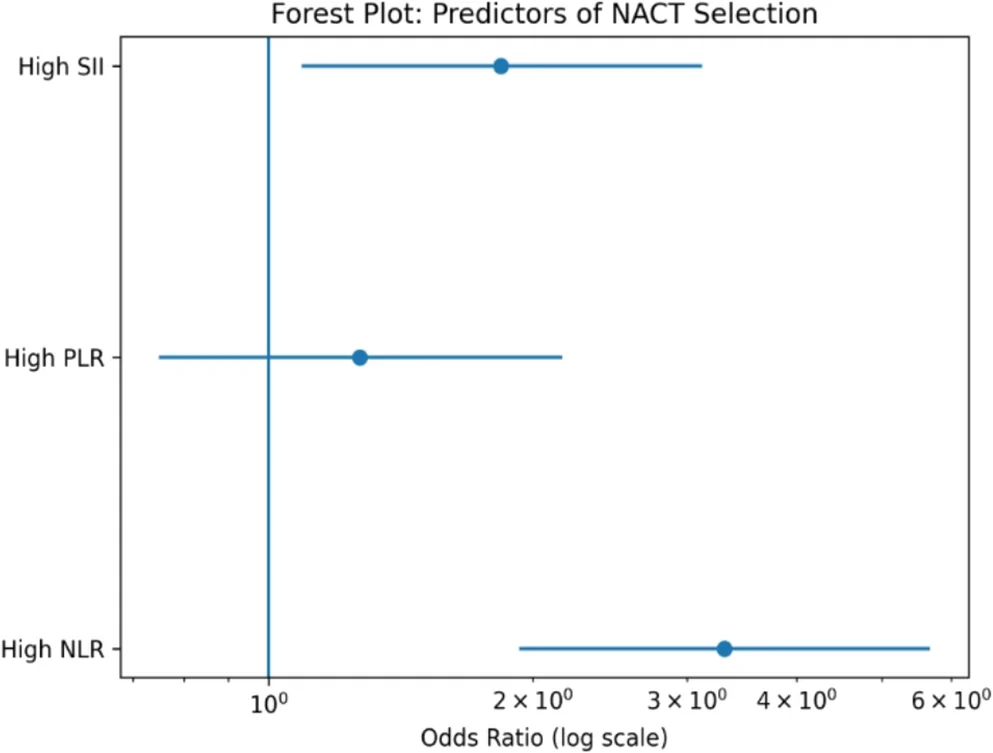
Forest plot of multivariable logistic regression analysis showing systemic inflammatory markers associated with the selection of neoadjuvant chemotherapy (NACT)

Multivariable logistic regression analyses evaluating factors associated with NACT selection are summarized in Table 2 and illustrated in Fig. 1. Due to collinearity among inflammatory markers, separate models were constructed for NLR, PLR, and SII, each adjusted for age, ECOG performance status, FIGO stage, and CA-125 level. High NLR (≥ 4.30) was independently associated with a significantly increased likelihood of NACT selection (OR 3.31, 95% CI 1.93–5.68, *p* < 0.001). High SII (≥ 1350) was also independently associated with NACT selection (OR 1.84, 95% CI 1.09–3.12, *p* = 0.023). In contrast, high PLR (≥ 222) was not significantly associated with treatment choice (OR 1.27, 95% CI 0.75–2.16, *p* = 0.369).

**Table 2:** Multivariable logistic regression analysis of factors associated with the selection of neoadjuvant chemotherapy (NACT)

	Odds ratio	95% confidence interval	*p* value
NLR			
High NLR (≥ 4.30)	3.31	1.93–5.68	**< **0.001
Age (per year)	1.02	1.00–1.05	0.076
ECOG (per unit)	1.02	0.65–1.62	0.919
FIGO stage (IV vs. III)	1.61	0.64–4.02	0.311
CA-125	1.00	0.99–1.00	0.628
PLR			
High PLR (≥ 222)	1.27	0.75–2.16	0.369
Age (per year)	1.02	1.00–1.05	0.051
ECOG (per unit)	1.02	0.66–1.58	0.938
FIGO stage (IV vs. III)	1.42	0.59–3.41	0.439
CA-125	1.00	0.99–1.00	0.827
SII			
High SII (≥ 1350)	1.84	1.09–3.12	0.023
Age (per year)	1.02	1.00–1.05	0.059
ECOG (per unit)	1.01	0.65–1.57	0.974
FIGO stage (IV vs. III)	1.44	0.59–3.50	0.423
CA-125	1.00	0.99–1.00	0.977

Abbreviations**:** *NACT *neoadjuvant chemotherapy, *OR *odds ratio, *CI *confidence interval,  *ECOG *Eastern Cooperative Oncology Group, *FIGO *International Federation of Gynecology and Obstetrics, *NLR*, neutrophil-to-lymphocyte ratio, *PLR *platelet-to-lymphocyte ratio, *SII *systemic immune-inflammation index, *CA-125*  cancer antigen 125

### Surgical outcomes and treatment strategy

**Fig. 2:**
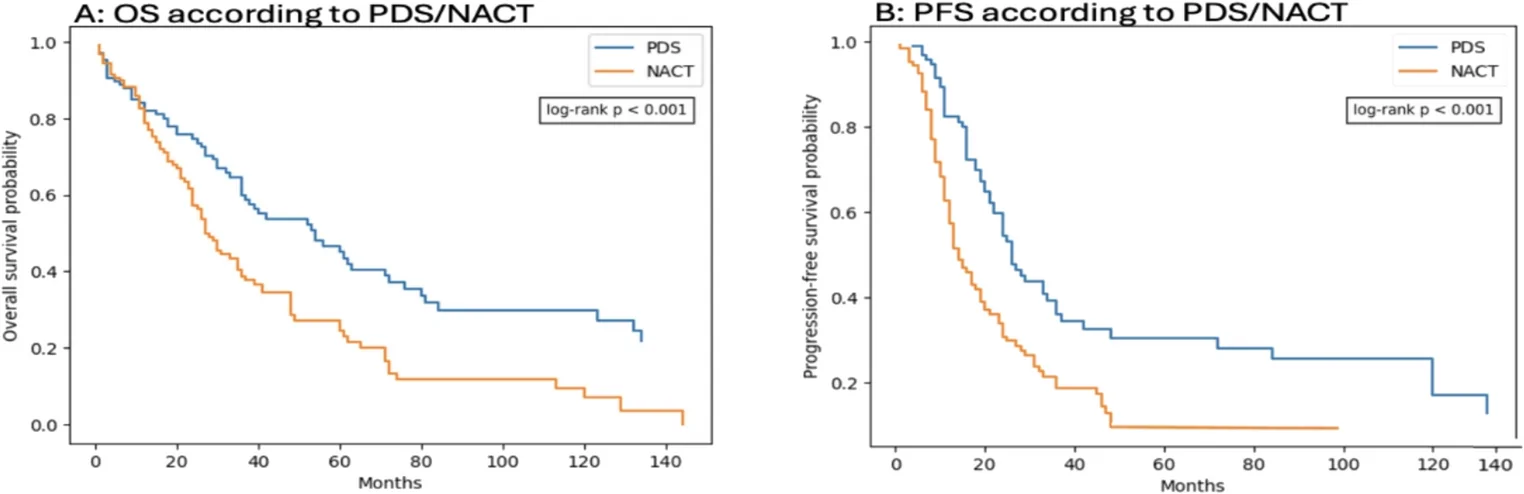
Survival according to treatment strategy. (**A**) Overall survival (OS) in patients treated with primary debulking surgery (PDS) versus neoadjuvant chemotherapy (NACT). (**B**) Progression-free survival (PFS) according to treatment strategy

Postoperative residual disease status is reported in Table 1. Rates of no visible residual disease were similar between the PDS and NACT groups (73.1% vs. 69.5%, *p* = 0.630), indicating comparable surgical outcomes once patients were selected for operative management. Survival analyses comparing PDS and NACT are depicted in Fig. 2 and summarized in Table 3. In univariable Cox regression analysis, NACT was associated with significantly worse OS (HR 1.95, 95% CI 1.42–2.68, *p* < 0.001) and PFS (HR 1.88, 95% CI 1.38–2.56, *p* < 0.001). After multivariable adjustment, treatment strategy remained an independent predictor of adverse outcomes. NACT was independently associated with inferior OS (HR 1.73, 95% CI 1.24–2.42, *p* = 0.001) and PFS (HR 1.77, 95% CI 1.25–2.51, *p* = 0.001).

**Table 3:** Univariable and multivariable cox regression analyses for OS and PFS

Variables	Univariable HR 95% CI	*P* Value	Multivariable HR 95% CI	*P* value
Overall Survival				
Age (per year)	1.04 (1.02–1.06)	**< **0.001	1.03 (1.02–1.05)	**< **0.001
FIGO stage (IV vs. III)	2.80 (1.75–4.48)	**< **0.001	2.45 (1.46–4.13)	**< **0.001
ECOG (per unit)	1.42 (1.08–1.87)	0.012	1.18 (0.94–1.50)	0.161
Treatment (NACT vs. PDS)	1.95 (1.42–2.68)	< 0.001	1.73 (1.24–2.42)	0.001
Residual disease (*≤* 1 cm vs. none)	1.31 (0.97–1.78)	0.081	—	—
High NLR (≥ 4.30 vs.<4.30)	1.67 (1.14–2.46)	0.009	1.51 (0.91–2.53)	0.113
High PLR (≥ 222 vs.<222)	1.42 (1.02–1.99)	0.041	1.23 (0.89–1.70)	0.219
High SII (≥ 1350 vs.<1350)	1.55 (1.08–2.23)	0.018	1.24 (0.89–1.72)	0.207
Progression Free Survival				
Age (per year)	1.03 (1.01–1.05)	0.002	1.02 (1.01–1.04)	0.005
FIGO stage (IV vs. III)	1.61 (0.92–2.82)	0.096	—	—
ECOG (per unit)	1.49 (1.14–1.95)	0.004	1.31 (1.01–1.71)	0.040
Treatment (NACT vs. PDS)	1.88 (1.38–2.56)	**< **0.001	1.77 (1.25–2.51)	0.001
Residual disease (*≤* 1 cm vs. none)	1.28 (0.96–1.71)	0.091	—	—
High NLR (≥ 4.30 vs.<4.30)	1.39 (1.00–1.94)	0.048	1.27 (0.93–1.74)	0.132
High PLR (≥ 222 vs.<222)	1.33 (0.97–1.83)	0.072	—	—
High SII (≥ 1350 vs.<1350)	1.41 (1.02–1.95)	0.039	1.19 (0.85–1.66)	0.320

Abbreviations**:** *OS *overall survival, *PFS  *progression-free survival,  *HR *hazard ratio, *CI* confidence interval, *ECOG *Eastern Cooperative Oncology Grou,  *FIGO *International Federation of Gynecology and Obstetrics, *PDS *primary debulking surgery,  *NACT *neoadjuvant chemotherapy, *NLR  *neutrophil-to-lymphocyte ratio, *PLR *platelet-to-lymphocyte ratio, *SII* systemic immune-inflammation index

### Prognostic impact of inflammatory markers on survival

Univariable Cox regression analyses (Table 3) demonstrated that high NLR, PLR, and SII were associated with worse OS and PFS. For OS, high NLR (HR 1.67, *p* = 0.009), high PLR (HR 1.42, *p* = 0.041), and high SII (HR 1.55, *p* = 0.018) were all significant in univariable analyses. However, in multivariable models adjusted for age, FIGO stage, ECOG performance status, and treatment strategy, none of the inflammatory markers retained independent prognostic significance. For OS, high NLR (HR 1.51, *p* = 0.113), high PLR (HR 1.23, *p* = 0.219), and high SII (HR 1.24, *p* = 0.207) were not statistically significant. Similar results were observed for PFS. In univariable analysis, high NLR (HR 1.39, *p* = 0.048) and high SII (HR 1.41, *p* = 0.039) were associated with worse PFS, whereas PLR showed a non-significant trend (*p* = 0.072). None of these markers remained significant in multivariable PFS models.

### Kaplan–Meier survival analyses

**Fig. 3:**
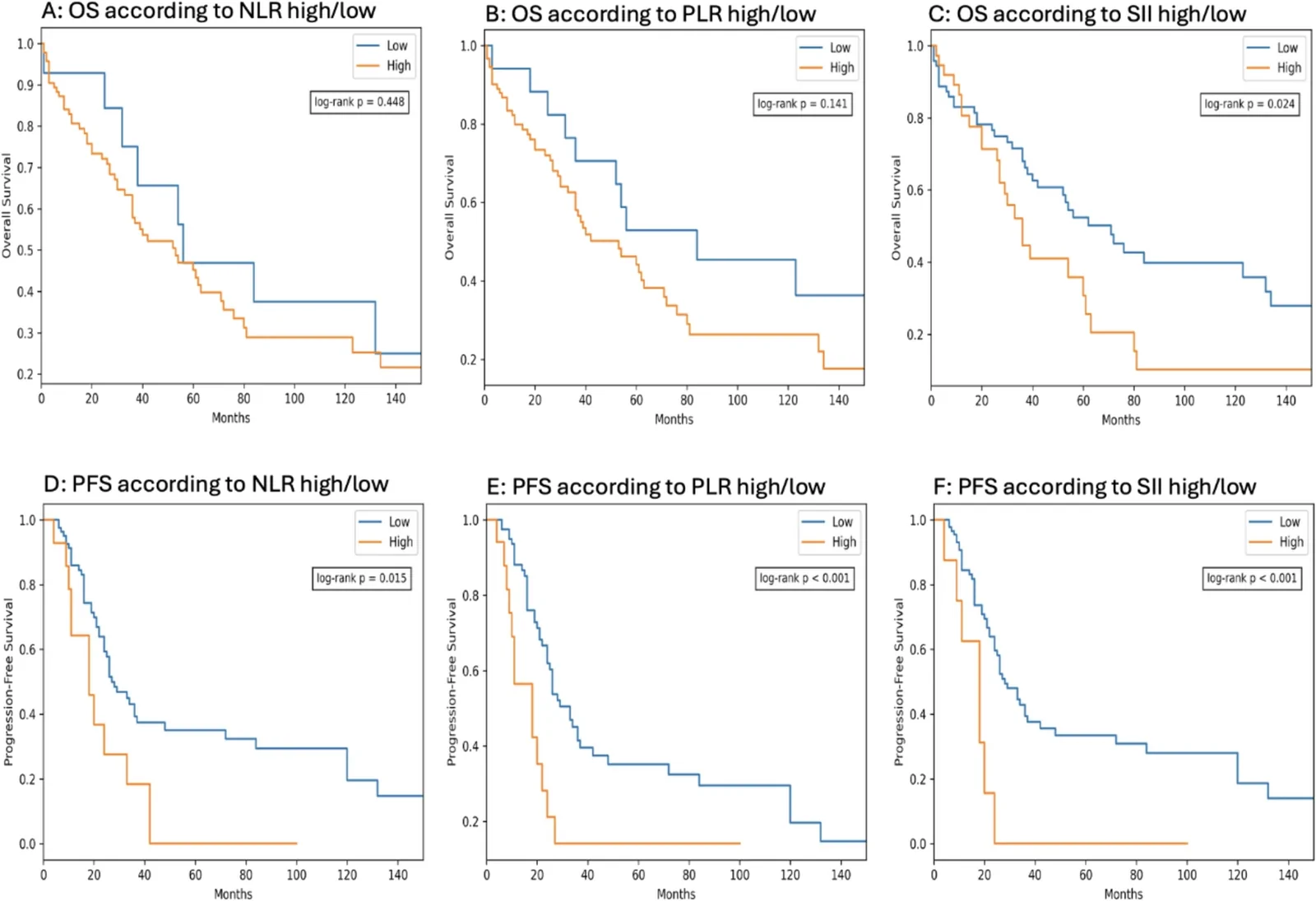
Overall survival and progression-free survival according to systemic inflammatory markers in patients undergoing primary debulking surgery. (**A**, **D**) Neutrophil-to-lymphocyte ratio (NLR), (**B**, **E**) platelet-to-lymphocyte ratio (PLR), and (**C**, **F**) systemic immune-inflammation index (SII)

**Fig. 4:**
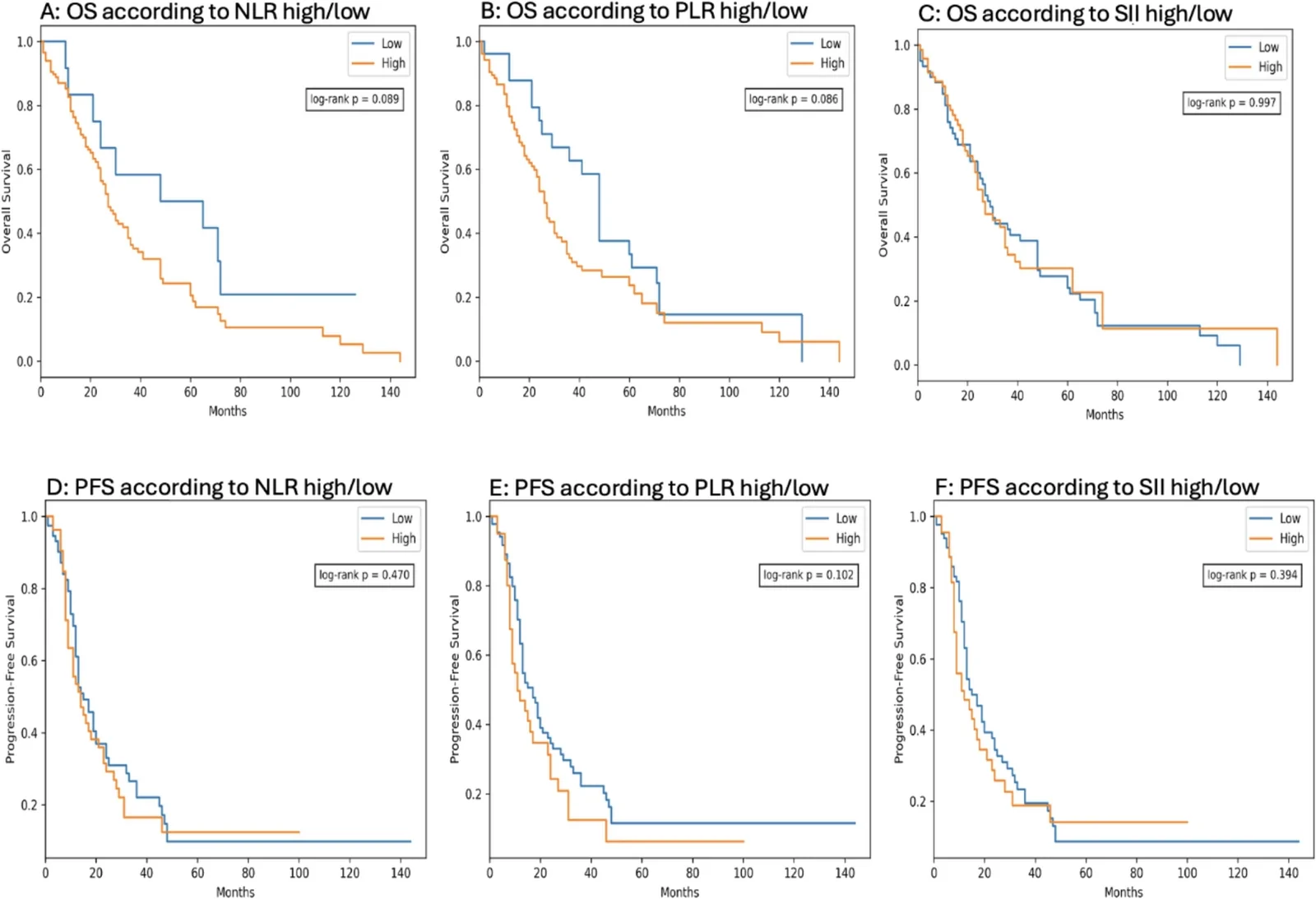
Overall survival and progression-free survival according to systemic inflammatory markers in patients treated with neoadjuvant chemotherapy. (**A**, **D**) Neutrophil-to-lymphocyte ratio (NLR), (**B**, **E**) platelet-to-lymphocyte ratio (PLR), and (**C**, **F**) systemic immune-inflammation index (SII)

For Kaplan–Meier survival analyses, inflammatory markers were dichotomized using predefined median cut-off values from the entire cohort (NLR ≥ 4.30, PLR ≥ 222, and SII ≥ 1350). The same cut-off values were applied to both the PDS and NACT groups to ensure consistency and avoid subgroup-specific optimization. Kaplan–Meier survival curves stratified by treatment strategy demonstrated significantly improved OS and PFS in patients undergoing PDS compared with those receiving NACT (Fig. 2, log-rank *p* < 0.001 for both). Subgroup Kaplan–Meier analyses stratified by inflammatory markers showed that elevated NLR, PLR, and SII significantly stratified survival in the PDS group (Fig. 3), whereas no statistically significant survival discrimination according to inflammatory marker status was observed in the NACT group (Fig. 4).

## Discussion

In this study, we evaluated the role of preoperative systemic inflammatory markers in patients with advanced-stage serous epithelial ovarian cancer, with a particular focus on their association with treatment selection and survival outcomes. Our findings demonstrate that elevated inflammatory markers—most notably NLR and SII—are strongly associated with the selection of neoadjuvant chemotherapy, whereas treatment strategy itself, rather than inflammatory status, independently determines overall and progression-free survival.

One of the key findings of this study is that patients selected for NACT exhibited a significantly higher systemic inflammatory burden at baseline. Elevated NLR, PLR, and SII values were consistently observed in the NACT group, and multivariable analyses confirmed that high NLR and SII independently increased the likelihood of receiving NACT. These results suggest that systemic inflammation reflects underlying disease burden and biological aggressiveness, factors that influence clinical decision-making regarding upfront surgery versus NACT. This observation aligns with previous studies demonstrating associations between inflammatory markers and advanced tumor spread, extensive peritoneal disease, and reduced likelihood of optimal cytoreduction [4, 22, 23].

High NLR emerged as the strongest independent predictor of NACT selection in our cohort. NLR is considered a surrogate marker of the balance between neutrophil-mediated pro-tumor inflammation and lymphocyte-dependent anti-tumor immune response [14, 15]. Elevated neutrophil levels may promote tumor progression through the secretion of cytokines, growth factors, and angiogenic mediators, while lymphopenia reflects impaired antitumor immunity [12]. Our findings support the hypothesis that a neutrophil-dominant inflammatory profile is closely linked to clinical assessments that favor neoadjuvant treatment strategies.

In contrast to their clear association with treatment allocation, inflammatory markers did not retain independent prognostic significance for OS or PFS after multivariable adjustment. While high NLR, PLR, and SII were associated with worse survival in univariable analyses, these effects were attenuated when established clinical factors and treatment strategy were taken into account. These findings suggest that systemic inflammatory markers primarily reflect underlying disease burden rather than acting as independent determinants of survival. Similar observations have been reported in prior studies, where the prognostic impact of inflammatory indices diminished after adjustment for tumor stage, residual disease, or treatment modality [24–26]. This finding may be explained by a mediation effect through treatment strategy, as elevated inflammatory markers were strongly associated with the likelihood of receiving neoadjuvant chemotherapy, which itself independently predicted survival outcomes. Accordingly, the apparent prognostic impact of systemic inflammation may be indirect, operating through its influence on treatment allocation rather than exerting a direct effect on survival.

Treatment strategy itself emerged as a dominant determinant of survival outcomes. Patients treated with NACT experienced significantly worse OS and PFS compared with those undergoing PDS, even after multivariable adjustment. Although randomized trials have demonstrated non-inferiority of NACT followed by interval debulking surgery compared with primary surgery in selected populations [7, 8], real-world data consistently show inferior outcomes among patients receiving NACT, likely reflecting higher baseline tumor burden, poorer functional status, and more aggressive disease biology [1, 9, 10]. These findings should be interpreted with caution, as treatment allocation was not randomized but based on clinical decision-making, introducing the potential for confounding by indication. Therefore, the observed association between treatment strategy and survival should not be interpreted as causal. Our findings reinforce the importance of careful patient selection and suggest that systemic inflammation may serve as a useful adjunctive marker in this process.

Subgroup Kaplan–Meier analyses further highlighted the context-dependent prognostic relevance of inflammatory markers. In patients undergoing PDS, elevated inflammatory markers were able to stratify survival outcomes, whereas no meaningful survival discrimination was observed among patients treated with NACT. This divergence may be explained by the relative homogeneity of the NACT population, in whom high disease burden and systemic inflammation are already prevalent, thereby limiting the discriminatory power of inflammatory markers. In contrast, among patients selected for PDS, inflammatory markers may capture biologic heterogeneity that influences postoperative outcomes.

The strengths of this study include its focus on a homogeneous population of patients with serous epithelial ovarian cancer, exclusion of non-serous histologies, and comprehensive evaluation of inflammatory markers in relation to both treatment selection and survival. Furthermore, the use of multivariable models and separate analyses for collinear inflammatory indices enhances the robustness of our findings.

Several limitations should be acknowledged. From a methodological perspective, careful selection of variables in multivariable models is critical for valid interpretation. Although residual disease is a well-established prognostic factor, it was not included in the primary models to avoid overadjustment, as it represents a post-treatment variable influenced by tumor burden and treatment strategy and may introduce bias, thereby obscuring the independent effects of baseline predictors. This approach is consistent with previous studies demonstrating that adjustment for treatment-related variables may attenuate the prognostic impact of baseline factors in ovarian cancer [25, 26]. The retrospective design introduces the potential for selection bias and unmeasured confounding. Inflammatory markers were measured at a single preoperative time point and may be influenced by transient conditions not fully captured in medical records. The study was conducted at a single center, which may limit generalizability. Although institutional practices were relatively consistent, the extended study period (2010–2025) may have introduced temporal heterogeneity related to evolving surgical techniques, shifting indications for neoadjuvant chemotherapy, and advances in supportive care, potentially affecting treatment patterns and outcomes. Finally, inflammatory markers were dichotomized using cohort-specific median values, which, although methodologically practical, are data-driven and may not reflect clinically established thresholds, potentially limiting generalizability and direct clinical applicability.

Despite these limitations, our results have important clinical implications. From a clinical perspective, systemic inflammatory markers may be incorporated into multimodal decision-making algorithms to support treatment selection in advanced ovarian cancer. In particular, elevated NLR and SII may help identify patients with a higher tumor burden or more aggressive disease biology who are less likely to benefit from upfront surgery. When interpreted alongside radiologic findings, performance status, and, where available, laparoscopic assessment scores, these readily accessible biomarkers may improve risk stratification and assist in selecting between primary debulking surgery and neoadjuvant chemotherapy. However, these markers should not be used in isolation, and their integration into clinical practice requires prospective validation.

In conclusion, preoperative systemic inflammation is closely associated with the selection of neoadjuvant chemotherapy in advanced serous epithelial ovarian cancer. While inflammatory markers reflect tumor burden and influence treatment allocation, long-term survival outcomes are primarily driven by treatment strategy rather than inflammatory status itself. Prospective studies are warranted to validate these findings and to explore whether integration of inflammatory markers into multimodal decision-making algorithms may improve individualized treatment planning.

## Data Availability

In view of the confidentiality and privacy of the participants, the datasets and other supporting documents are available from the corresponding author upon reasonable request.
